# LongTerm Outcomes of Three-Port Laparoscopic Right Hemicolectomy *Versus* Five-Port Laparoscopic Right Hemicolectomy: A Retrospective Study

**DOI:** 10.3389/fonc.2021.762716

**Published:** 2021-09-30

**Authors:** Tao Zhang, Yaqi Zhang, Xiaonan Shen, Yi Shi, Xiaopin Ji, Shaodong Wang, Zijia Song, Xiaoqian Jing, Feng Ye, Ren Zhao

**Affiliations:** ^1^ Department of General Surgery, Ruijin Hospital, Shanghai Jiao Tong University School of Medicine, Shanghai, China; ^2^ Department of GI, Ruijin Hospital, Shanghai Jiao Tong University School of Medicine, Shanghai, China; ^3^ Yueyang Hospital of Integrated Traditional Chinese and Western Medicine, Shanghai University of Traditional Chinese Medicine, Shanghai, China

**Keywords:** colon cancer, laparoscopy, longterm outcomes, three-port laparoscopic right hemicolectomy, five-port laparoscopic right hemicolectomy, three-port laparoscopic assisted colectomy

## Abstract

**Purpose:**

The aim of this study is to compare the long-term outcomes of three-port laparoscopic right hemicolectomy (TPLRC) and five-port laparoscopic right hemicolectomy (FPLRC) with retrospective analysis.

**Methods:**

A total of 182 patients who accepted laparoscopic right hemicolectomy with either three ports (86 patients) or five ports (96 patients) from January 2012 to June 2017 were non-randomly selected and analyzed retrospectively.

**Results:**

More lymph nodes were harvested in the TPLRC group than in the FPLRC group [17.5 (7), 14 (8) ml, *p* < 0.001]. There was less blood loss in the TPLRC group [50 (80) *vs*. 100 (125) ml, *p* = 0.015]. There were no significant differences in the other short-term or oncological outcomes between the two groups. The overall survival and disease-free survival were equivalent.

**Conclusions:**

TPLRC is recommendable as it guarantees short- and long-term equivalent outcomes compared with FPLRC.

## Introduction

It has been three decades since Jacobs reported the first laparoscopic right hemicolectomy of the world in 1991 (1). The application of laparoscopic technology in colonic surgery has gradually become acceptable, even recommended. Multiple large-scale clinical studies (2–8) had proven its safety, feasibility, and equilibrium to laparotomy in oncological outcome and survival. These studies had made scientific judgment of laparoscopic technology and provided novel variations of laparoscopic surgery, such as single-incision laparoscopic surgery (SILS), port-reduced laparoscopic surgery, and so on. It was not until the year 2011 when, at almost the same time, a study on three-port laparoscopic colectomy of 24 patients was reported by Dr. Park from Korea (9) and a 49-case study was published by Dr. I. Seow-En from Singapore (10). After that, some small-scale clinical short-term outcomes of three-port laparoscopic colectomy, scattered in Egypt, Italy (11), and elsewhere, had been reported. Our center had published our short-term outcomes of three-port laparoscopic right hemicolectomy (TPLRC) *versus* five-port laparoscopic right hemicolectomy (FPLRC) in 2020, involving 168 patients and using a propensity score matching study, in which TPLRC presented non-inferiority to FPLRC (12). Years later, the long-term outcome of our study is to be described in this article.

## Materials and Methods

### Patients and Collection

A retrospective chart review was performed on 182 patients with right hemi-colonic adenocarcinoma who received either TPLRC or FPLRC from January 2012 to June 2017 at Ruijin Hospital, Shanghai Jiao Tong University School of Medicine in China. Patients who were older than 80 upon receiving surgery, with stage IV colonic cancer concomitant with other malignant tumors, and with incomplete records for review were excluded. An approval of the study was granted by the Institutional Review Board of our hospital, and informed consent forms for the operations, from all patients, were recorded.

All the operations were performed by the same well-experienced surgeon who had completed more than 1,500 laparoscopic colorectal surgeries. A total of 86 patients who underwent TPLRC and 96 patients who went through a FPLRC were enrolled in this study.

The clinicopathologic information and perioperative outcomes were reviewed, including age, sex, body mass index, the American Society of Anesthesiologists grade, previous abdominal surgery, tumor site, operation time, estimated blood loss, time to cereal diet, length of postoperative hospital stay, postoperative complications, tumor size, number of harvested lymph nodes, proximal and distal resection margins, specimen length, lymph node metastasis, and pathologic stage according to the 8th edition of the AJCC Cancer Staging Manual. The postoperative complications were graded according to the Clavien–Dindo classification.

Follow-up surveillance was conducted in accordance with the National Comprehensive Cancer Network (NCCN) guidelines. Recurrence was confirmed by radiological or histological methods. Patients in stage III or stage II with high risk were routinely sent to chemotherapy for further treatment according to the guideline. For patients who presented with metastasis, a second-line chemotherapy was carried out, and standard evaluation was made to confirm whether surgery would be necessary.

### Surgical Procedure

#### Position

The patients were placed in a lithotomy, Trendelenburg position. After the pneumo-peritoneum was established, the operation table was tilted to the left as appropriate to provide better exposure. In TPLRC, the surgeon was to the left of the patient, while the cameraman positioned between the legs of the patient. In FPLRC, the assistant was on the right side of the patient.

#### Trocar Placement

**Figure 1:**
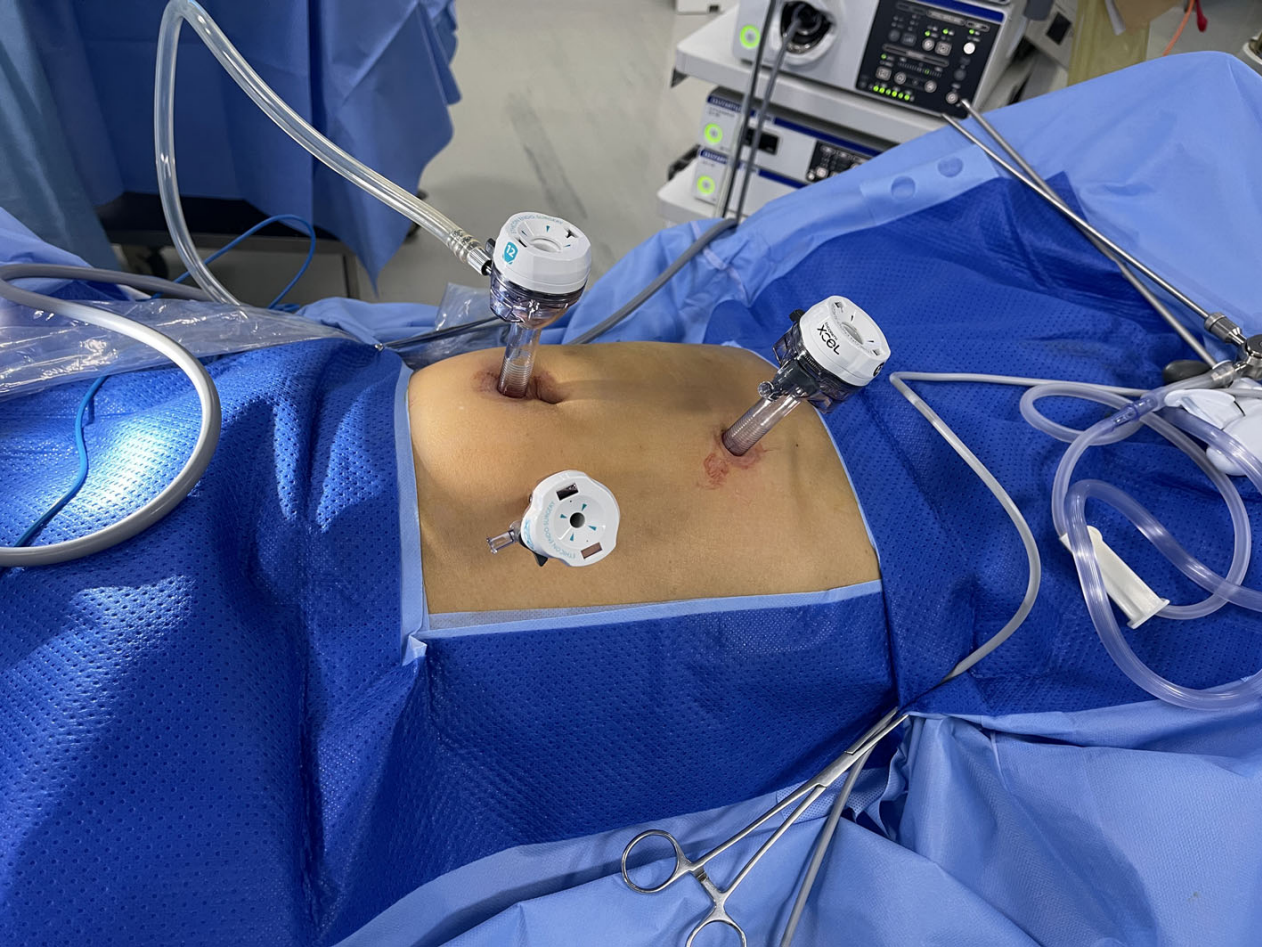
Trocar placement.

A trans-umbilical 12-mm port was inserted for the camera, and two others (5 and 12 mm) were placed at the left mid-clavicular line in TPLRC. There were two more ports in FPLRC, placed at the right mid-clavicular line or in another appropriate position (see **Figure 1**).

**Figure 2:**
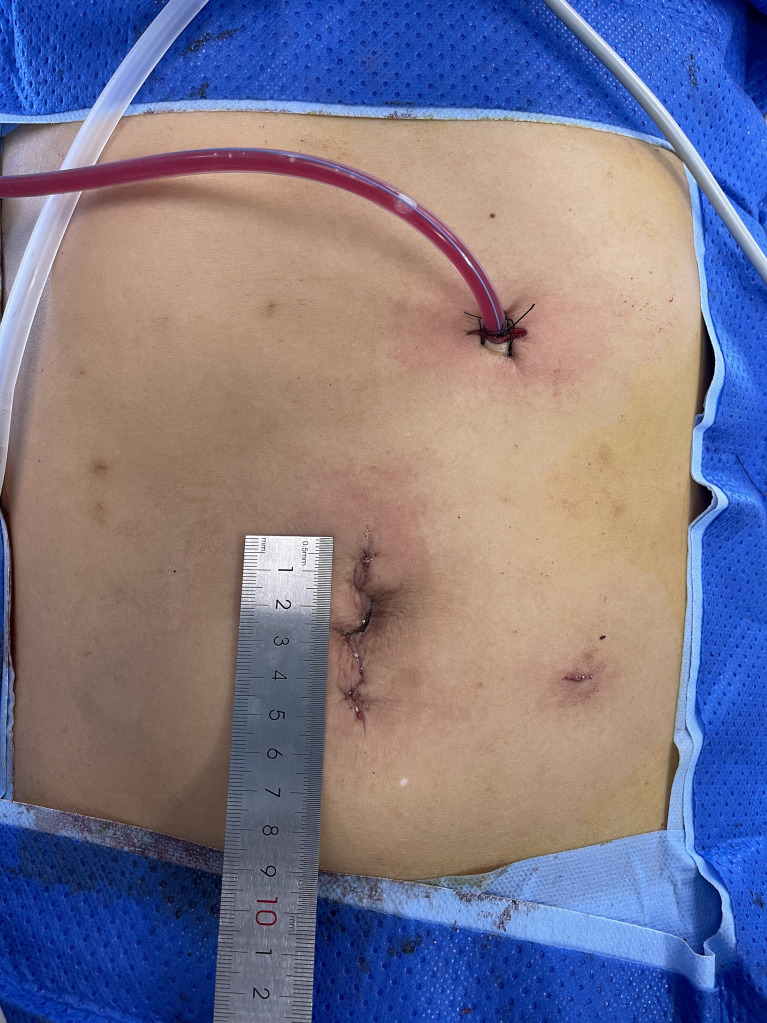
Incisions.

**Figure 3:**
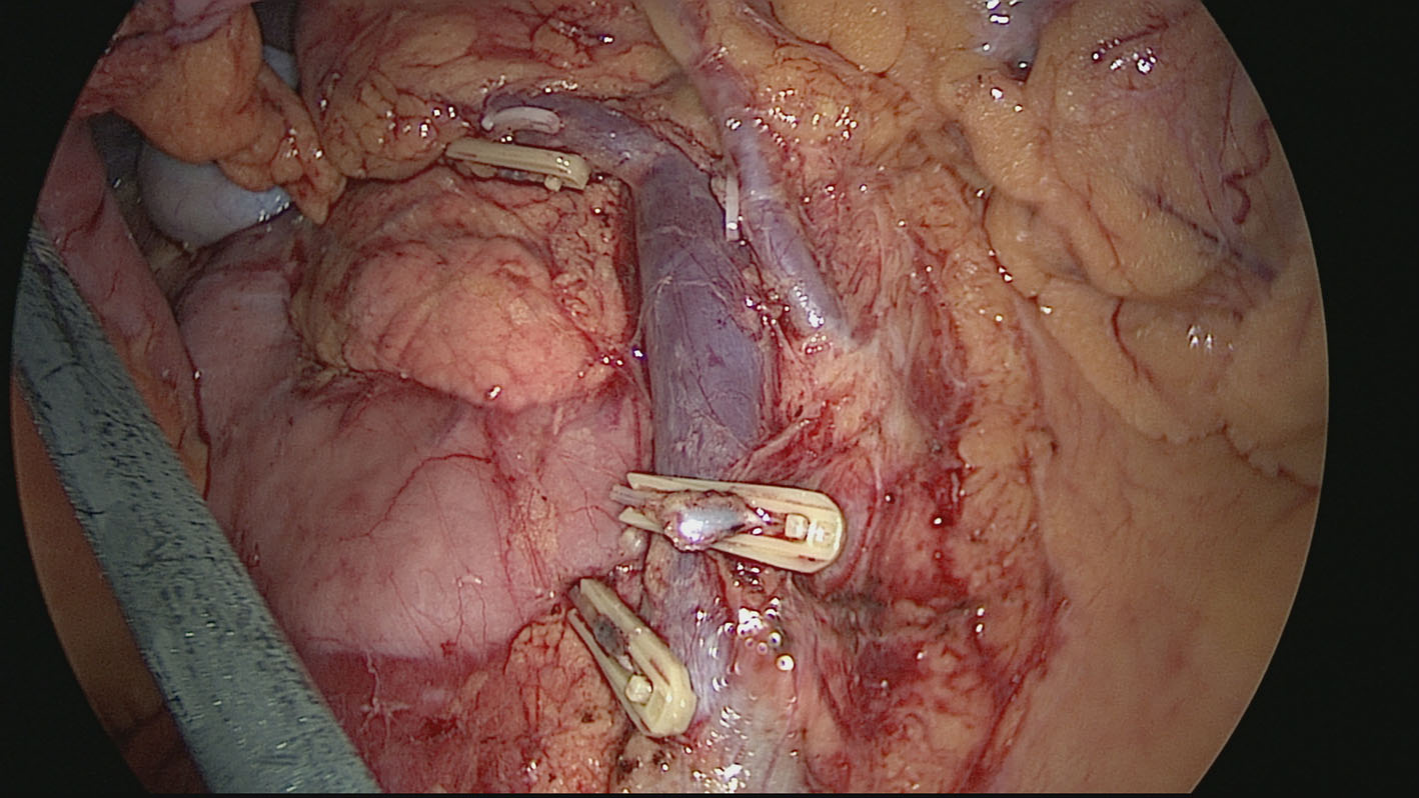
A vessel-priority D3 dissection + CME.

A vessel-priority D3 dissection + CME was carried out intracorporeally. The specimen was retrieved through an arc umbilical incision, with the wound protected (see **Figure 2**). Resection and anastomosis were performed extracorporeally by hand sutures or with a stapler (see **Figure 3**).

### Statistical Analysis

Statistical analysis was performed with SPSS (version 23.0, SPSS Inc., Chicago, IL, USA). The statistically significant differences were evaluated using Mann–Whitney *U*-test, Student’s *t*-test, *χ* ^2^ test, and Fisher exact test as appropriate. The overall survival and disease-free survival rates were estimated using the Kaplan–Meier method and compared using the log-rank test. A *p-*value <0.05 was considered statistically significant.

## Results

### Baseline Characteristics

There was no significant difference between the two groups in the baseline variables (see **Table 1**).

**Table 1:** Baseline characteristics.

Variable	Three-port (*N* = 86)	Five-port (*N* = 96)	Statistics	*P*-value
Age (year)				
Median (IQR)	62 (17)	66 (13)	*Z* = -1.174	0.240
Sex				
Male (%)	41 (47.7)	46 (47.9)	*χ* ^2^ = 0.001	0.974
Female (%)	45 (52.3)	50 (52.1)		
Body mass index				
<18.50 (%)	7 (8.1)	3 (3.1)	*χ* ^2^ = 0.003	0.954
18.50–24.99 (%)	61 (70.9)	60 (62.5)		
>24.99 (%)	18 (20.9)	33 (34.4)	*χ* ^2^ = 5.487	0.064
American Society of Anesthesiologists score				
I, II (%)	69 (80.2)	80 (83.3)	*χ* ^2^ < 0.001	
III, IV (%)	17 (19.8)	16 (16.7)	*χ* ^2^ = 0.294	0.701
Previous abdominal surgery (%)	25 (29.1)	33 (34.4)	*χ* ^2^ = 0.588	0.524
Tumor site				
Ileocecus (%)	21 (24.4)	34 (35.4)		
Ascending colon (%)	33 (38.4)	21 (21.9)		
Hepatic flexure (%)	24 (27.9)	33 (34.4)		
Transverse colon (%)	8 (9.3)	8 (8.3)	*χ* ^2^ = 6.631	0.085

### Intraoperative and Perioperative Outcomes

In the TPLRC group, less blood loss [50 (80) *vs*. 100 (125) ml, *p* = 0.015] was observed. There was no significant statistical difference between TPLRC and FPLRC with respect to operative time, length of postoperative hospital stay, time to cereal diet, and postoperative complications. Besides these, no case was converted to laparotomy, and there was neither readmission nor mortality within 30 days of surgery. There were three cases of grade III and IV complications in the three-port group: one case of anastomotic leakage which needed an intraperitoneal wash and two cases of cardiac dysfunction due to a very severe preoperative heart disease and who thus went to the intensive care unit after surgery. These patients were discharged afterwards with normal diet and New York Heart Association I–II (see **Table 2**). However, the distribution of complications showed no statistical difference in each group.

**Table 2:** Intraoperative and perioperative outcomes.

Variable	Three-port (*N* = 86)	Regular (*N* = 96)	Statistics	*P*-value
Operation time (min)				
Median (IQR)	140.0 (45.0)	150.0 (52.5)	*Z* = -1.861	0.063
Estimated blood loss, (ml)				
Median (IQR)	50.0 (80.0)	100.0 (125.0)	*Z* = -2.431	**0.015**
Postoperative hospital stays (days)				
Median (IQR)	9.0 (3.0)	9.0 (2.0)	*Z* = -0.845	0.398
Time to anal exhaust				
Median (IQR)	4.0 (2.0)	4.0 (2.0)	*Z* = 0.297	0.766
Time to liquid diet (days)				
Median (IQR)	6.0 (2.0)	5.0 (2.0)	*Z* = -0.310	0.976
Grade of complications				
0–I (%)	73 (84.9)	90 (92.8)		
II (%)	10 (11.6)	6 (6.3)		
III (%)	1 (1.2)	0 (0)		
IV (%)	2 (2.3)	0 (0)	*χ* ^2^ = 5.239	0.155

In bold: A p-value <0.05 was considered statistically significant.

### Pathologic and Oncologic Outcomes

More lymph nodes were harvested in the TPLRC group than in the FPLRC group [17.5 (7), 14 (8) ml, *p* < 0.001]. There were more T1 and T2 patients in the FPLRC group. The tumor size, proximal and distal resection margins, histology, metastatic lymph node, and TNM stage manifested no difference between the TPLRC and FPLRC groups (see **Table 3**).

**Table 3:** Pathologic and oncologic outcomes.

Variable	Three-port (*N* = 86)	Five-port (*N* = 96)	Statistics	*P*-value
Tumor size (cm)				
Median (IQR)	4.0 (2.0)	4.5 (2.0)	*Z* = -0.658	0.510
Specimen length (cm)				
Median (IQR)	27.0 (9.0)	28.0 (9.0)	*Z* = -0.403	0.687
Proximal resection margins, cm				
Median (IQR)	12.0 (8.0)	10.0 (7.3)	*Z* = -1.914	0.056
Distal resection margins, cm				
Median (IQR)	11.25 (8.0)	12.25 (10.5)	*Z* = -0.815	0.415
Lymph node harvest				
Median (IQR)	17.5 (7)	14 (8)	*Z* = -4.152	**<0.001**
Positive LN				
Median (IQR)	0 (1)	0 (1)	*Z* = -0.544	0.586
TNM stage				
I (%)	3 (3.4)	11 (11.3)	*χ* ^2^ = 6.085	0.193
II (%)	50 (7.5)	52 (53.6)		
III (%)	33 (37.9)	33 (34.0)		
T stage				
1, 2 (%)	3 (3.5)	15 (15.6)	*Z*	0.954
3, 4 (%)	83 (96.5)	81 (84.4)	*χ* ^2^ = 7.498	**0.006**
N stage				
0 (%)	52 (60.5)	63 (65.6)	*χ* ^2^ = 0.003	0.954
1 (%)	28 (32.6)	23 (24.0)	*χ* ^2^ = 0.003	0.954
2 (%)	6 (7.0)	10 (10.4)	*χ* ^2^ = 1.99	0.368

In bold: A p-value <0.05 was considered statistically significant.

### DFS and OS

In this study, the follow-up period ranges from 1 to 108 months. Eight patients (9.3%) in the TPLRC group and 13 patients (13.5%) in the FPLRC group were lost during the follow-up period (*χ* ^2^ = 0.799, *P* = 0.371). The median follow-up period was 72 months (95% CI, 68.89–75.11) in the TPLRC group and 82 months (95% CI, 72.92–91.08) in the FPLRC group (*χ* ^2^ = 2.837, *p* = 0.092).

**Figure 4:**
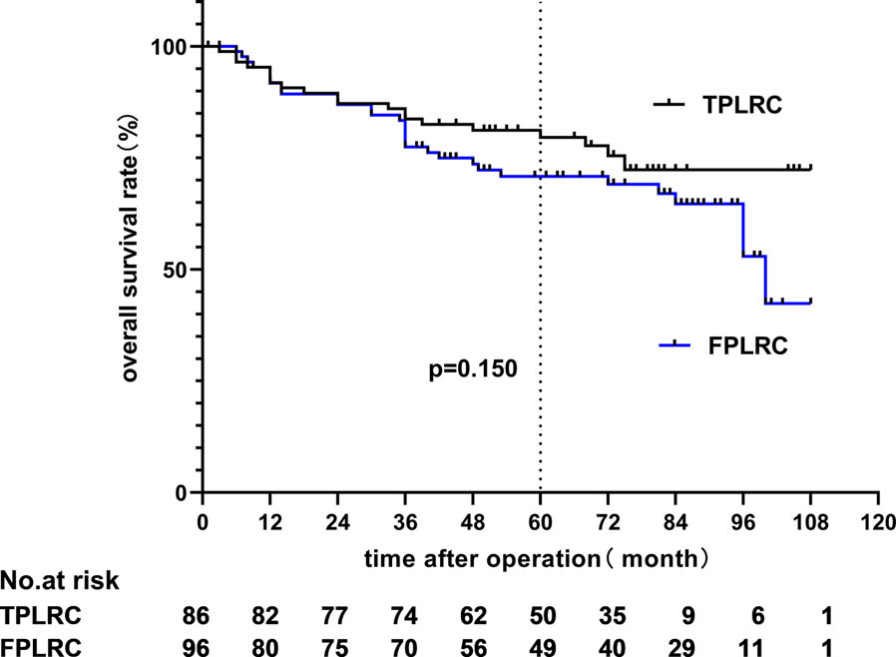
Overall survival rate.

**Figure 5:**
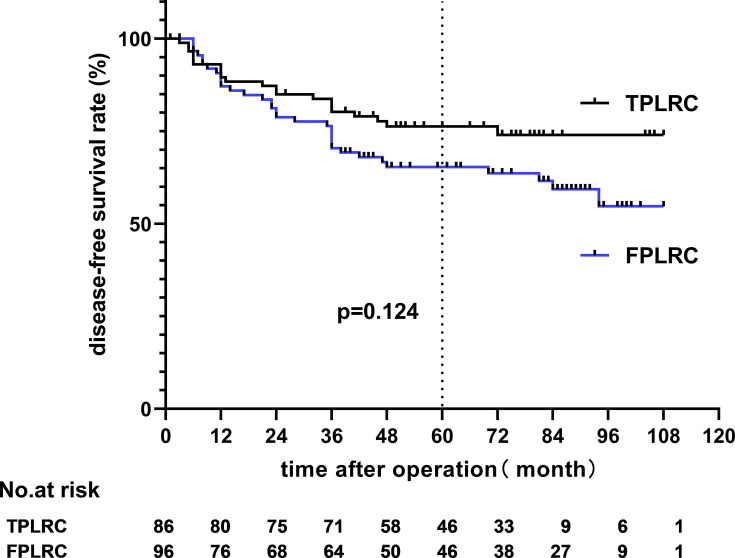
Disease-free survival rate.

The 5-year overall survival (OS) rates of the TPLRC and FPLRC groups were 79.6 and 70.9% (hazard ratio, HR: 0.642; 95% CI: 0.348–1.185; *p* = 0.150), respectively, and the 5-year disease-free survival (DFS) rates were 65.3 and 76.3% (HR, 0.646; 95% CI, 0.366–1.139; *p* = 0.124), respectively (see **Figures 4** and **5**).

Since the longest follow-up time reached 9 years, we also compared the 9-year OS and DFS rates of the two groups, and the results showed no statistical significance (*p* = 0.208 and *p* = 0.099).

## Discussion

Broadly speaking, the idea of laparoscopy can be traced back to ancient Rome. One of the inventors of the encyclopedia, a famous Roman doctor, Aulus Cornelius Celsus, first reported to have drained “evils humor” by using percutaneous devices (now called trocars) (8, 13). Since then, particularly in the 20th century, the invention of a lighting system, telescopes, and insufflators and the integrity of all laparoscopic devices made the ancient dreams come true (14, 15). The first laparoscopic cholecystectomy (LC) of the world was performed by Mühe in the mid-1980s (13, 16, 17). After that, all varieties of laparoscopic-assisted surgery embraced its boom, and it would not take another 2000 years to make a leap and bounce in laparoscopic surgery.

Nowadays, laparoscopic surgery had confirmed its equality in safety and feasibility, better cosmetic outcome, and quicker recovery when compared with open surgery. A conventional laparoscopic colorectal surgery usually requires five or more trocars to insert instruments. With the increase in the number of trocars, the concomitant problems would increase accordingly. Postoperative incisional pain, incision-related wound infection, bleeding, hernia, or metastasis are not rare (18–20). Besides these, during the operation, unskilled assistants would sometimes interfere with the surgeon and even cause iatrogenic injury due to the so-called off-screen effect or violent tractions (21). The earliest reported clinical study about three-port laparoscopic colectomy dated back to 2011 (9, 10), and it is believed that many other surgeons had completed this kind of operations even earlier. We began to carry out three-port laparoscopic colectomy in 2012 in order to conquer the problem of shortage in surgical assistants. This point of view was identified with Jung Ryul Oh et al. (22) to carry out this operation with one surgeon and one cameraman. In this study, three-port surgery brags an advantage in less blood loss and more lymph nodes harvested. The other perioperative and oncological outcomes presented no difference between the three-port and five-port groups. The distribution in postoperative complication presented no difference between the TPLRC and FPLRC groups. Although the intention of port-reduced laparoscopic surgery was to reduce port-related complications and others, there was one anastomotic leakage in the three-port group. The patient was concomitant with systemic lupus erythematosus with a long-time intake of glucocorticoid. It reminds us that careful selection of patients should be conducted preoperatively for the three-port surgery. Did the port-reduced surgery bring less pain to the patients compared to a conventional laparoscopic one? We have already denied the pain-ease theory in our previous article (12). Whether the cosmetic effect of port-reduced surgery can aesthetically please the patients is not reported in any of the present articles.

The clinical study about three-port laparoscopic-assisted colectomy (TLAC) is rarely reported. All we can search till now are retrospective analyses with short-term outcomes, in which TLAC possessed similar peri-operative and oncological outcomes as the five-port laparoscopic-assisted colectomy. In this study, we reviewed the laparoscopic right hemi-colectomy records and found out the long-term survival results. The average follow-up period exceeded 5 years, with the longest surveillance reaching 9 years in our study. OS and DFS manifested no difference between the TPLRC and FPLRC groups. Although there was no statistical difference between the 9-year OS, the separation of the curves is visible. Nine patients in the three-port group died from liver metastasis, two with lung metastasis and one with bone metastasis. Thirteen patients in the five-port group died from liver metastasis, two with lung metastasis and one with peritoneal metastasis. The main reason behind the difference may be the selection bias, and we cannot deny that there might have been an intention to choose some patients with earlier stage of the disease at the very beginning of the surgery, while the baseline of the two groups of patients presents no difference with statistical significance. Despite the controversy, this is hitherto the one and only long-term outcome ever reported, which can be an evidential support to recommend this kind of operation.

Geisler et al. (23) believe that the accumulation of TLAC is conducive to the development of single-incision surgery. Gash et al. (24) believe that the key to mastering single-incision laparoscopic colorectal surgery is developing TLAC surgery. Our center began the attempt of single-incision laparoscopic colectomy in December 2013. Passing the learning curve in SILS for sigmoid colon and upper rectum in our center took approximately 11 cases. Kirk et al. (25) reported 70 consecutive cases of SILS right hemi-colectomy, comparing the operation time, estimated bleeding, complications, and pathological results between the groups. Finally, about 40 cases were determined to pass through LC. Haas et al. (26) reported about 30 to 36 cases of LC that can pass through the SILS of the right colon by the cumulative summation method. The essence of SILS is to move multiple ports to the same position; therefore, the development of three-port laparoscopic surgery is helping to shorten the LC of single-incision surgery. This point of view is consistent with the report of Haas et al. (26). However, due to the lack of a standardized comparison, the value of three-port surgical practice in shortening LC in single-incision surgery needs to be further evaluated.

This study has its limitation. The data was retrospectively collected, so selection bias could not be avoided, and the sample scale in this study is relatively not enough. In order to conquer the defect of a retrospective analysis, we have already conducted a single-center randomized control trial for this kind of operation.

## Conclusions

In conclusion, TPLRC had an advantage in terms of less blood loss and more lymph nodes harvested and is non-inferior to FPLRC in all other variables, especially OS and DFS. Besides these, this kind of operation requires two trocars less than the conventional one, and only one surgeon and one cameraman were needed, which makes it worthy to recommend to experienced surgeons.

## Data Availability Statement

The raw data supporting the conclusions of this article will be made available by the authors, without undue reservation.

## Ethics Statement

The studies involving human participants were reviewed and approved by a single center randomized controlled study of three-port laparoscopy and conventional laparoscopy assisted colorectal resection. The patients/participants provided their written informed consent to participate in this study.

## Author Contributions

RZ and TZ had full access to all of the data in the study and take responsibility for the integrity of the data and the accuracy of the data analysis. Concept and design: TZ. Acquisition, analysis, or interpretation of data: All authors. Drafting of the manuscript: TZ, YZ, and XS. Critical revision of the manuscript for important intellectual content: Statistical analysis: TZ and YZ. Obtained funding: RZ, XQJ, and TZ. Administrative, technical, or material support: All authors. Supervision: FY and RZ. All authors contributed to the article and approved the submitted version.

## Funding

This study was supported by the National Natural Science Foundation of China [81902374 (XQJ)], the Shanghai Hospital Development Center [16CR2064B (RZ)], the Shanghai Municipal Health Construction Commission [201540026 (RZ)], and the Youth Development Program of Ruijin Hospital, Shanghai Jiaotong University School of Medicine [2019QNPY01010 (TZ)].

## Conflict of Interest

The authors declare that the research was conducted in the absence of any commercial or financial relationships that could be construed as a potential conflict of interest.

## Publisher’s Note

All claims expressed in this article are solely those of the authors and do not necessarily represent those of their affiliated organizations, or those of the publisher, the editors and the reviewers. Any product that may be evaluated in this article, or claim that may be made by its manufacturer, is not guaranteed or endorsed by the publisher.
